# Prediction of ESRD and Death Among People With CKD: The Chronic Renal Impairment in Birmingham (CRIB) Prospective Cohort Study

**DOI:** 10.1053/j.ajkd.2010.07.016

**Published:** 2010-12

**Authors:** Martin J. Landray, Jonathan R. Emberson, Lisa Blackwell, Tanaji Dasgupta, Rosita Zakeri, Matthew D. Morgan, Charlie J. Ferro, Susan Vickery, Puja Ayrton, Devaki Nair, R. Neil Dalton, Edmund J. Lamb, Colin Baigent, Jonathan N. Townend, David C. Wheeler

**Affiliations:** 1 Clinical Trial Service Unit and Epidemiological Studies Unit, University of Oxford, Oxford, UK; 2 Queen Elizabeth Hospital, University Hospitals (Birmingham) NHS Foundation Trust, Edgbaston, Birmingham, UK; 3 School of Immunity and Infection, College of Medical and Dental Sciences, University of Birmingham, Birmingham, UK; 4 Department of Clinical Biochemistry, East Kent Hospitals University NHS Trust, Kent and Canterbury Hospital, Canterbury, UK; 5 Department of Chemical Pathology, Barnet and Chase Farm Hospitals NHS Trust, Barnet General Hospital, Barnet, UK; 6 Department of Biochemistry, Royal Free Hospital, London, UK; 7 WellChild Laboratory, King's College London, Evelina Children's Hospital, London, UK; 8 Centre for Nephrology, University College London Medical School, Royal Free Campus, London, UK

**Keywords:** Chronic kidney disease, risk prediction, outcomes, death, end-stage renal disease

## Abstract

**Background:**

Validated prediction scores are required to assess the risks of end-stage renal disease (ESRD) and death in individuals with chronic kidney disease (CKD).

**Study Design:**

Prospective cohort study with validation in a separate cohort.

**Setting & Participants:**

Cox regression was used to assess the relevance of baseline characteristics to risk of ESRD (mean follow-up, 4.1 years) and death (mean follow-up, 6.0 years) in 382 patients with stages 3-5 CKD not initially on dialysis therapy in the Chronic Renal Impairment in Birmingham (CRIB) Study. Resultant risk prediction equations were tested in a separate cohort of 213 patients with CKD (the East Kent cohort).

**Factors:**

44 baseline characteristics (including 30 blood and urine assays).

**Outcomes:**

ESRD and all-cause mortality.

**Results:**

In the CRIB cohort, 190 patients reached ESRD (12.1%/y) and 150 died (6.5%/y). Each 30% lower baseline estimated glomerular filtration rate was associated with a 3-fold higher ESRD rate and a 1.3-fold higher death rate. After adjustment for each other, only baseline creatinine level, serum phosphate level, urinary albumin-creatinine ratio, and female sex remained strongly (*P* < 0.01) predictive of ESRD. For death, age, N-terminal pro-brain natriuretic peptide, troponin T level, and cigarette smoking remained strongly predictive of risk. Using these factors to predict outcomes in the East Kent cohort yielded an area under the receiver operating characteristic curve (ie, C statistic) of 0.91 (95% CI, 0.87-0.96) for ESRD and 0.82 (95% CI, 0.75-0.89) for death.

**Limitations:**

Other important factors may have been missed because of limited study power.

**Conclusions:**

Simple laboratory measures of kidney and cardiac function plus age, sex, and smoking history can be used to help identify patients with CKD at highest risk of ESRD and death. Larger cohort studies are required to further validate these results.

In Western populations, approximately 5%-10% of the adult population have an estimated glomerular filtration rate (eGFR) <60 mL/min/1.73 m^2^ (ie, chronic kidney disease [CKD] stages 3-5).^1-3^ These individuals are at increased risk of death (particularly from cardiovascular causes) and progression to end-stage renal disease (ESRD; ie, the need for dialysis or kidney transplant) compared with those without CKD.^4,5^ Decreased kidney function is associated with many factors that might be associated with increased risks of death, ESRD, or both.^6-11^ However, for a particular patient with CKD, it is unclear how knowledge of phenotypic factors can be used to determine the likelihood of requiring renal replacement therapy or of dying within the next few years.^12^ In particular, risk equations developed in the general population (such as those derived for cardiovascular death from the Framingham study)^13^ are not accurate in patients with CKD because the relationships between classic risk markers (such as cholesterol level or blood pressure) and adverse outcomes are weak and can even be reversed because of extensive confounding by both disease and treatment.^14^

This study aims to quantify and compare the risks of death and ESRD in a cohort of patients with CKD stages 3-5 (but not receiving renal replacement therapy) and develop clinically useful methods for predicting those that would be applicable to other populations with known kidney disease.

## Methods

Details of the design and methods of the Chronic Renal Impairment in Birmingham (CRIB) Study have been described previously^9-11^ and are summarized next.

### Recruitment and Eligibility Criteria

Between December 1997 and September 1999, individuals with a serum creatinine level >1.5 mg/dL (but not receiving renal replacement therapy) attending a single large UK renal clinic in Birmingham were invited to take part in the study. Approximately half the potentially eligible patients agreed to participate. There were no significant differences in age, sex, or kidney function between those who agreed and those who declined to participate. All participants provided written consent. Ethics approval was obtained from the local research ethics committee.

### Baseline Assessment

At the initial interview, research nurses recorded medical history, current medication, clinical measurements, and a 12-lead electrocardiogram. Urine and nonfasting blood samples were collected. Blood was separated using centrifugation (3,000*g* at 4°C for 15 minutes) within 60 minutes and stored at −80°C.

In addition to the assays described previously,^9-11^ N-terminal pro-brain natriuretic peptide (NT-pro-BNP) and troponin T were measured using immunoassay methods on Roche automated analyzers (Roche, www.roche.com). A high troponin level was defined as ≥0.01 μg/L.^15^ Symmetric dimethylarginine, asymmetric dimethylarginine, and arginine were assayed using stable isotope-dilution electrospray mass spectrometry on a SCIEX API4000 analyzer (Applied Biosystems, www.appliedbiosystems.com). Creatinine was measured using the Jaffé reaction, and eGFR was calculated using the 4-variable Modification of Diet in Renal Disease (MDRD) Study equation.^16^ Within- and between-batch coefficients of variation were <10% for all assays.

### Follow-up

Participants were flagged for mortality at the Office for National Statistics (United Kingdom), which provided the date and cause of all deaths up to July 1, 2006. The development of ESRD (ie, initiation of maintenance dialysis therapy or kidney transplant) was tracked through hospital and dialysis unit records to the end of 2007. For ESRD, participants who did not reach ESRD were censored at the date of death or the date at which they were last known to be alive and free of ESRD. For mortality, participants not known to have died by July 1, 2006, were censored on that date.

### Statistical Methods

#### Relevance of Individual Characteristics to Risk

When appropriate, baseline characteristics were normalized by applying a log transformation. Cox proportional hazards regression was used to estimate the average age- and sex-adjusted relevance of each baseline characteristic to the risks of ESRD and death (the small numbers of participants with missing data were assigned the mean or median value, as appropriate). The magnitude of improvement in risk prediction (compared with a model containing only age and sex) was estimated by twice the change in the log-likelihood statistic (which, under the null hypothesis of no improvement, gives a χ^2^ statistic with 1 *df*). This test (the likelihood ratio test) provides not only a statistical test for improvement in fit, but also a quantitative measure of the extent to which the added term improves risk prediction.^17^ For continuous exposures, log-linearity was assessed by testing the statistical significance of including a quadratic term into the model (other tests for nonlinearity were not performed). To avoid overfitting in the subsequent multivariable risk prediction models, only characteristics statistically significant at *P* < 0.01 were considered further.

#### Combined Relevance of Several Characteristics to Risk

Characteristics found to be predictive of risk at the *P* < 0.01 significance level in age- and sex-adjusted models were entered simultaneously (together with age and sex) into a single model. A backwards elimination approach with a strict *P* < 0.01 inclusion criterion was then used to obtain an optimal subset of variables (a forwards selection approach yielded the same results). Possible interactions between individual factors were not considered, and no restrictions were placed on age and sex to remain in the final model. As before, the difference in twice the log-likelihood between 2 nested models (which gives a χ^2^ statistic with *df* equal to the difference in the number of variables between models) was used to provide both an assessment of how well the reduced set of factors predicted risk compared with the full set and a formal test for improvement in model fit. Relative risks (RRs; estimated using hazard ratios from the Cox model) associated with differences in this final subset of characteristics were calculated and used to derive absolute risk prediction equations. To assess whether these average relative risks seen during the follow-up period might vary in magnitude during follow-up, the proportional hazards assumption of the Cox model was tested by examination of the Schoenfeld residuals.^18^

#### External Validation

External validation of the final prediction equations for ESRD and death was performed using information available from a separate cohort of 213 patients with stages 3-5 CKD recruited at a renal unit in East Kent between June 2003 and June 2004. Participants were followed up for ESRD (mean, 2.6 years) and death (mean, 3.3 years).^19^ Identical methods were used to measure NT-pro-BNP and troponin T, but urinary albumin-creatinine ratio was not measured. External validation was performed by applying the frozen regression coefficients from the final CRIB models to the individual risk factors for each patient in the independent East Kent cohort, yielding a predicted risk of ESRD and death for each patient in this external cohort. For each outcome, the Hanley-McNeil method^20^ was used to calculate the area under the receiver operating characteristic curve (ie, AUROC or C statistic, a measure of the discriminatory ability of the risk model) and its 95% confidence interval (CI). Calibration was examined by separating individuals into 5 equal-sized groups based on their predicted risk of each outcome and comparing the observed annual event rates in these 5 groups with the predicted average annual event rates from the Cox model (using the baseline hazards seen in CRIB). Kaplan-Meier survival curves were also produced for each of 3 equal-sized predicted risk groups to illustrate the associations with observed risk over time.

## Results

### Baseline Characteristics of Study Population

The CRIB cohort included 382 participants with stages 3-5 CKD: 88 had stage 3 CKD (mean eGFR, 37.0 mL/min/1.73 m^2^), 178 had stage 4 CKD (mean eGFR, 21.9 mL/min/1.73 m^2^), and 116 had stage 5 CKD (mean eGFR, 10.1 mL/min/1.73 m^2^. Baseline characteristics of the cohort are listed in Table 1, and results of laboratory assays performed on baseline samples are listed in Table 2.

### Risk of ESRD and All-Cause Mortality

**Figure 1:**
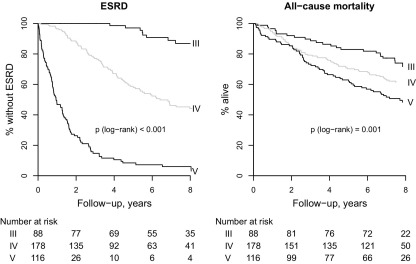
Time to end-stage renal disease (ESRD) and death in the Chronic Renal Impairment in Birmingham (CRIB) Study, by initial chronic kidney disease (CKD) stage. The *P* value for the log-rank test corresponds to the test of equal survival across all 3 baseline CKD groups. CKD stage was as defined by the National Kidney Foundation's K/DOQI (Kidney Disease Outcomes Quality Initiative) Work Group in 2002: stage 3, estimated glomerular filtration rate (eGFR) of 30-60 mL/min/1.73 m^2^; stage 4, eGFR of 15-30 mL/min/1.73 m^2^; and stage 5, eGFR <15 mL/min/1.73 m^2^. eGFR was calculated using the 4-variable Modification of Diet in Renal Disease (MDRD) Study equation. Individuals receiving renal replacement therapy at baseline (either dialysis or a kidney transplant) were excluded from the study.

During a mean of 4.1 years' (1,571 person-years) follow-up for renal events, 190 participants reached ESRD (mean rate, 12.1% per annum [pa]; Table 3). In participants initially at CKD stages 3, 4, and 5, annual event rates of ESRD were 1.6%, 9.6%, and 58.2%, respectively (between-group comparison, *P* < 0.001; Fig 1). No patients with stage 3 CKD at baseline required dialysis or transplant within the first 4 years of follow-up. In those with stage 4 CKD initially, median time to ESRD was about 5 years longer than for those with stage 5 CKD (∼6 vs 1 year, respectively; Fig 1).

During a mean of 6.0 years' (2,302 person-years) follow-up for mortality, 150 participants died (mean rate, 6.5% pa; Table 3). Of the 190 patients who reached ESRD, the subsequent mortality rate was much greater in the 143 patients who received dialysis (71 subsequent deaths [8.3% pa]) than in the 47 patients who received a kidney transplant (2 subsequent deaths [0.5% pa]; *P* < 0.001). Mortality rates were higher in participants with more advanced CKD at baseline: In those initially at CKD stages 3, 4, and 5, annual mortality rates were 3.9%, 6.3%, and 9.2%, respectively (between-group comparison, *P* = 0.001; Fig 1).

**Figure 2:**
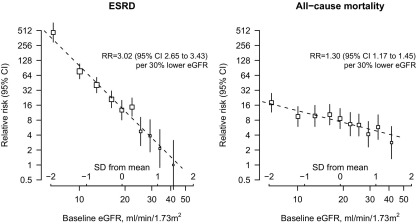
Age- and sex-adjusted relative risk (RR) of end-stage renal disease (ESRD) and death in the CRIB (Chronic Renal Impairment in Birmingham) Study by baseline estimated glomerular filtration rate (eGFR; calculated using the 4-variable Modification of Diet in Renal Disease [MDRD] Study equation). Both the horizontal and vertical axes are shown on a logarithmic scale. The points in the right hand panel have been adjusted so that the absolute mortality rates they represent are comparable with the absolute ESRD rates represented in the left hand panel (thus, the point at which the 2 lines cross is the level of eGFR above which, in the CRIB cohort, the risk of death started to exceed the risk of ESRD). Abbreviation: CI, confidence interval.

There were strong inverse log-linear associations between proportional differences in eGFR and risks of ESRD and death (Fig 2). This was much more marked for ESRD than for death. Within the range of eGFRs studied, each 30% lower baseline eGFR (eg, 40 vs 28 mL/min/1.73 m^2^) was associated with an approximately 3-fold increase in risk of ESRD (RR, 3.02; 95% CI, 2.65-3.43; *P* < 0.001) and a 1.3-fold increase in risk of death (RR, 1.30; 95% CI, 1.17-1.45; *P* < 0.001). In this cohort, the risk of dying during follow-up exceeded that of developing ESRD for those with eGFR >25 mL/min/1.73 m^2^ (Fig 2).

### Individual Risk-Relations With ESRD and Mortality

Of the 44 baseline characteristics assessed, 20 showed strong (ie, *P* < 0.01) associations with ESRD independently of age and sex (Table S1, available as online supplementary material), of which 18 were still significant at *P* < 0.001. For all-cause mortality, 19 baseline characteristics showed strong associations independently of age and sex (Table S2), of which 12 were still significant at *P* < 0.001.

### Risk Models for ESRD and All-Cause Mortality

The baseline characteristics identified as associated with ESRD in age- and sex-adjusted analyses (listed in Table S1), including age and sex, were considered for potential inclusion in an ESRD risk model. Using a backwards elimination selection process (see statistical methods), 4 independently informative predictors were identified: creatinine level, phosphate level, urinary albumin-creatinine ratio, and female sex (Table 4). Together, these predicted risk to a degree similar to all 22 candidate variables in combination (on dropping the other 18 factors, χ^2^ for model fit decreased by only 26 [from 379.6 to 353.6], which, when tested against a χ^2^ distribution with 18 *df*, gives *P =* 0.10). Creatinine level alone provided >80% of the information (χ^2^_1_ = 308.5) available from knowledge of all 22 candidate variables. In the final model, a 50% higher baseline serum creatinine concentration (about 1 SD [standard deviation]) was on average associated with an RR for ESRD of 3.25 (95% CI, 2.69-3.92; *P* < 0.001; Table 5) during follow-up. However, this RR decreased in magnitude with increasing follow-up. The RR of ESRD for a 50% higher baseline creatinine level was about twice as strong in the first 2 years of follow-up (by which time about half the ESRD events had been observed: RR, 4.71 [95% CI 3.52-6.30]) than in subsequent years (RR, 2.43 [95% CI 1.90-3.10]). In contrast, there was no evidence of such nonproportionality for the other predictors of ESRD. Given the overall average relevance of baseline creatinine level to risk, the RR of ESRD was about 50% higher for a 30% higher phosphate level (RR, 1.46; 95% CI, 1.21-1.77; *P* = 0.001) and for a 5-fold higher urinary albumin-creatinine ratio (RR, 1.51; 95% CI, 1.24-1.85; *P* < 0.001), which represent differences of about 1 SD, and with female sex (RR, 1.54; 95% CI, 1.13-2.09; *P* = 0.006; Table 5). Similar estimates for these other factors were obtained when the RR associated with creatinine level was specifically modeled as a time-dependent covariate.

For mortality, 4 baseline characteristics (of the 21 candidate exposures, including age and sex; see Table S2) were identified as independent predictors: NT-pro-BNP level, age, current cigarette smoking, and increased troponin T level (χ^2^ for combined model fit = 138.8 compared with 160.8 if all 21 factors were included; a difference of 22, which, when tested against a χ^2^ distribution with 17 *df*, gives *P =* 0.2; Table 4). Of these, age and NT-pro-BNP level were related most strongly to risk. In the final model, the risk of death was approximately doubled for each 15 years of older age (RR, 1.95; 95% CI, 1.54-2.45; *P* < 0.001), each 5-fold higher NT-pro-BNP level (RR, 1.72; 95% CI, 1.41-2.12; *P* < 0.001), current cigarette smoking (RR, 2.36; 95% CI, 1.56-3.59; *P* < 0.001), and a positive troponin T result (RR, 1.83; 95% CI, 1.26-2.66; *P* = 0.001; Table 5). There was no evidence that these RRs varied in magnitude with increasing duration of follow-up (global test for nonproportionality, *P* = 0.2).

These independently informative predictors were used to develop risk equations for the prediction of ESRD (based on creatinine level, phosphate level, urinary albumin-creatinine ratio, and sex) and death (based on NT-pro-BNP level, age, cigarette smoking, and troponin T level). These equations have been incorporated in an open-access risk calculator, available at www.ctsu.ox.ac.uk/cribcalculator.

### External Validation of Risk Prediction Equations

**Figure 3:**
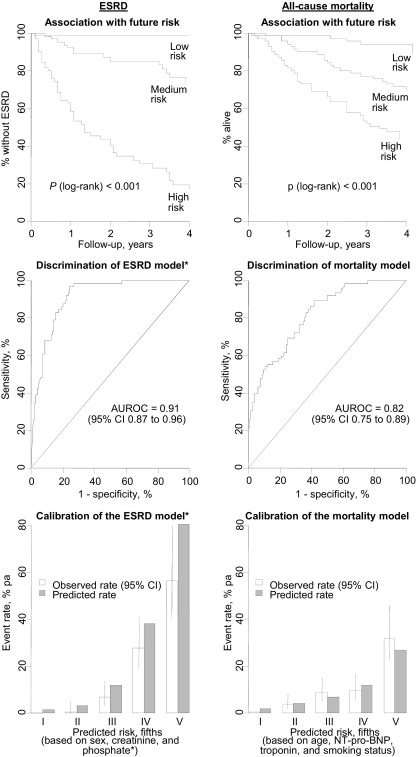
External validation of the CRIB (Chronic Renal Impairment in Birmingham) risk score equations in the East Kent cohort: Kaplan-Meier survival curves by third of the predicted risk distributions (top panels); receiver operating characteristic curves (middle panels); and observed versus predicted annual event rates (bottom panels). The *P* value for the log-rank test corresponds to the test of equal survival across all 3 predicted risk groups. *Urinary albumin-creatinine ratio was not available in the East Kent cohort; therefore, the measures of discrimination and calibration shown reflect the ability of the other factors (sex, creatinine level, and phosphate level) to predict end-stage renal disease (ESRD) risk. Abbreviations: AUROC, area under the receiver operating characteristic curve (C statistic); CI, confidence interval; NT-pro-BNP, N-terminal pro-brain natriuretic peptide.

Within the East Kent cohort of 213 patients with CKD stages 3-5 (not on renal replacement therapy), baseline characteristics were broadly similar to those in the CRIB Study (see Table S3). Prediction equations derived from the CRIB cohort were assessed independently for discrimination and calibration in the East Kent cohort (because urinary albumin-creatinine ratio was not measured in the East Kent cohort, all participants were assigned an arbitrary value of 350 mg/g). Even without knowledge of ACR, there was clear separation in risk over time between people at low, medium, and high risk of each outcome (Fig 3A), and the AUROC (the C statistic) was very good for both ESRD (0.91; 95% CI, 0.87-0.96) and death (0.82; 95% CI, 0.75-0.89; Fig 3B). When participants in the East Kent cohort were separated into 5 groups based on predicted risks of each outcome, observed annual event rates were systematically lower than predicted rates for ESRD, but reasonably well matched for mortality (Fig 3C). Virtually identical estimates of discrimination and calibration of ESRD risk were obtained when prediction equations that allowed the RR for creatinine level to vary during follow-up were used.

## Discussion

This study confirms the high risks of progression to ESRD and death in patients with stages 3-5 CKD (particularly those with eGFR <45 mL/min/1.73 m^2^) who had been referred to a specialist nephrology center, but were not receiving dialysis. The risks of each of these outcomes were closely associated with baseline kidney function throughout the range studied, but this association was much stronger for ESRD than for death. Within this cohort, the absolute risk of ESRD was greater than the risk of death for individuals with eGFR approximately <25 mL/min/1.73 m^2^ (ie, CKD stage 4 or 5), whereas the converse was true for those with better levels of baseline kidney function. However, the precise threshold at which this change in prognosis occurs is likely to vary both between populations and over time, largely depending on the underlying absolute risk of death.^5,21,22^

The finding that more advanced renal impairment is associated with increased risks of death and in particular ESRD is likely to be caused in part by the “horse-racing effect,”^23^ a term used to describe the observation that the absolute value of a risk exposure tends to correlate positively with the rate of change in that exposure. Thus, because baseline creatinine concentration is likely to correlate highly with the rate of decrease in an individual's kidney function, the excess risks observed in individuals for whom kidney function was worst at a single “baseline” assessment may occur because these people were experiencing progression most rapidly. (This also could explain why the RR of ESRD associated with differences in baseline creatinine level was higher in earlier years of follow-up than in the later years.) Nonetheless, this still does not diminish the utility of such a measurement as a simple prognostic tool.

The CRIB cohort is well characterized and includes a very large range of potential risk markers recorded at baseline. The principal determinant of the probability of progression to ESRD is kidney function. The final model includes 3 markers of kidney damage (serum creatinine level, serum phosphate level, and urinary albumin-creatinine ratio) plus sex, which may reflect that GFR is lower in women than men for a given creatinine level (and therefore the RR of ESRD for female sex is >1). Age did not remain in the final model because much of the predictive association between age and ESRD was provided by creatinine level alone. This is in contrast to other previously published risk scores, in which age remained significantly related to risk independently of creatinine level.^24,25^ In our study, creatinine level alone provided about 80% of the predictive information for ESRD that was provided by including a full array of 21 different factors (age, sex, and 19 other factors that were strongly predictive of ESRD in age- and sex-adjusted models), indicating the importance of the current level of kidney function in predicting the risk of progression to ESRD. Previous studies that have developed risk scores for kidney disease progression have also found that markers of kidney function (creatinine level, eGFR, and proteinuria) or factors highly correlated with kidney function (such as blood pressure, diabetes, anemia, and serum phosphate level) are particularly informative.^26-30^

In contrast, the probability of dying depends chiefly on markers of morbidity (ie, age, NT-pro-BNP level, troponin T level, and cigarette smoking). Increased plasma concentrations of NT-pro-BNP or troponin T (assays commonly available in routine clinical laboratories) provide quantitative estimates of the severity of cardiovascular disease in an individual: NT-pro-BNP level may be associated with left ventricular hypertrophy and dysfunction, fluid overload, and ischemia, although it also may indicate more severe decreases in GFR,^31^ and troponin T most likely reflects a combination of myocardial necrosis, hypertrophy, and dysfunction.^32^ Increased NT-pro-BNP level is a powerful predictor of mortality in individuals with vascular disease, diabetes, or heart failure, in the unselected general population, and in patients with CKD.^31,33-36^ Detectable levels of troponin T were predictive of all-cause and cardiac mortality in studies of elderly people in the general population^37^ and patients with moderate to severe CKD both before and after the need for dialysis therapy.^15,31,38-39^

Many candidate factors were measured in this study, but were not associated independently with renal progression or death in age- and sex-adjusted analyses. In patients with more severe renal impairment, relationships between exposures and risk of clinically important events are likely to be substantially confounded by either disease (eg, cholesterol level) or treatment (eg, blood pressure).^14^ As a consequence, prediction equations developed in the general population (largely without significant kidney dysfunction) perform very poorly in the CKD population.^13^ Many of the exposures measured correlated closely, particularly with serum creatinine level (Table S4), and thus do not appear in the final risk score models. Of course, these models provide no information about causality (or lack of it) and are intended to be predictive rather than explanatory.

Results of the external validation show excellent discrimination for ESRD and all-cause mortality (AUROCs, 0.91 and 0.82, respectively). Had urinary albumin-creatinine ratio been available, it is likely that discrimination would have been even better. Levin et al^40^ recently have reported risk equations for ESRD and death. Unfortunately, many of the baseline factors required for these equations were not available in the East Kent cohort. However, within the CRIB cohort, these equations yielded smaller C statistics of 0.65 (95% CI, 0.58-0.72) for ESRD and 0.73 (95% CI, 0.67-0.79) for death. When the CRIB mortality risk prediction equation was applied to the East Kent cohort for the outcome of death before ESRD, the C statistic (0.79; 95% CI, 0.71-0.86) was similar to that seen for all deaths. However, the study was too small to allow risk equations to be developed separately for specific causes of death, whereas a lack of follow-up for nonfatal cardiovascular events precluded further analyses of this type.

This study confirms the log-linear association between RR of both ESRD and death with proportional decreases in eGFR. Using simple currently available laboratory measures of kidney and cardiac function plus age, sex, and history of cigarette smoking, it is possible to identify patients with CKD at greatest absolute risk of these clinical outcomes within the next few years. We have developed an open-access web application that illustrates how these equations might be used in routine clinical practice, available at www.ctsu.ox.ac.uk/cribcalculator.

The results are most directly relevant to patients with eGFR <45 mL/min/1.73 m^2^, and further work is required to assess the performance of these equations in larger cohorts and individuals with less severe decreases in kidney function.

## Figures and Tables

**Table 1:** Baseline Characteristics of the CRIB Cohort

	All	CKD Stage at Baseline^a^		
		3	4	5
No. of people	382	88	178	116
Age (y)	61.5 ± 14.3	59.3 ± 14.6	62.8 ± 14.6	61.1 ± 3.3
eGFR^a^ (mL/min/1.73 m^2^)	21.8 ± 10.7	37.0 ± 5.9	21.9 ± 4.2	10.1 ± 2.8
Men (%)	64.9	83.0	60.7	57.8
Disease history (%)				
Vascular disease	44.8	50.0	41.6	45.7
Diabetes mellitus	17.3	17.0	14.6	21.6
Left ventricular hypertrophy^b^	20.0	12.8	21.2	23.9
Medication (%)				
Any antihypertensive	83.0	77.3	80.9	90.5
Aspirin	27.2	30.7	28.7	22.4
Vitamin D	35.3	9.1	28.1	66.4
Calcium	21.7	4.5	16.3	43.1
Iron	25.1	6.8	20.8	45.7
Erythropoietin	9.4	1.1	3.9	24.1
Folic acid/B vitamins	12.0	8.0	10.7	17.2
Smoking status (%)				
Never	36.6	31.8	38.2	37.9
Ex-smoker	50.8	54.5	50.0	49.1
Current	12.6	13.6	11.8	12.9
Ethnicity (%)				
White	88.0	85.2	90.4	86.2
Black	5.8	10.2	2.2	7.8
Asian	6.3	4.5	7.3	6.0
Physical measurements				
Weight (kg)	76.2 ± 16.6	81.6 ± 15.7	74.4 ± 14.8	74.7 ± 19.0
Height (m)	1.69 ± 0.09	1.71 ± 0.08	1.68 ± 0.09	1.68 ± 0.09
Waist-to-hip ratio	0.90 ± 0.11	0.91 ± 0.09	0.90 ± 0.09	0.91 ± 0.14
Body mass index (kg/m^2^)	26.6 ± 5.0	27.8 ± 5.1	26.3 ± 4.6	26.3 ± 5.5
SBP (mm Hg)	151.6 ± 22.4	151.3 ± 22.6	152.3 ± 23.9	150.9 ± 19.9
DBP (mm Hg)	83.9 ± 11.8	86.6 ± 12.0	84.5 ± 11.9	81.0 ± 11.1

*Note:* Values shown as number, percentage, or mean ± standard deviation for continuous measures. Missing data: left ventricular hypertrophy, 4%; weight, 1%; waist-to-hip ratio, 4%; body mass index, 1%; and blood pressure, 1%. Conversion factor for eGFR in mL/min/1.73 m^2^ to mL/s/1.73 m^2^, ×0.01667. Abbreviations: CKD, chronic kidney disease; CRIB, Chronic Renal Impairment in Birmingham; DBP, diastolic blood pressure; eGFR, estimated glomerular filtration rate; K/DOQI, Kidney Disease Outcomes Quality Initiative; MDRD, Modification of Diet in Renal Disease; SBP, systolic blood pressure.

a CKD stage was as defined by the National Kidney Foundation's K/DOQI Work Group in 2002: stage 3, eGFR of 30-60 mL/min/1.73 m^2^; stage 4, eGFR of 15-30 mL/min/1.73 m^2^; and stage 5, eGFR <15 mL/min/1.73 m^2^. eGFR was calculated using the 4-variable MDRD Study equation. Individuals receiving renal replacement therapy at baseline (either dialysis or a kidney transplant) were excluded from the study.

b Based on an electrocardiogram.

**Table 2:** Baseline Laboratory Values in the CRIB Cohort

	All	CKD Stage at Baseline^a^		
		3	4	5
No. of people	382	88	178	116
Kidney function				
Creatinine (mg/dL)	3.0 (2.2-4.6)	2.0 (1.8-2.1)	2.9 (2.5-3.3)	5.7 (4.9-6.7)
Cystatin C (mg/L)	3.0 (2.1-4.0)	1.8 (1.6-2.1)	2.8 (2.3-3.3)	4.4 (3.8-5.0)
UACR (mg/g)	460 (88-1,257)	250 (29-799)	339 (67-1,156)	896 (285-1,579)
Symmetric dimethylarginine (μmol/L)	1.38 (1.06-1.92)	0.95 (0.83-1.13)	1.36 (1.11-1.65)	2.25 (1.83-2.72)
Urea (mg/dL)	114 ± 54	65 ± 21	104 ± 35	168 ± 49
Urate (mg/dL)	7.5 ± 2.0	7.0 ± 1.8	7.7 ± 1.9	7.7 ± 2.2
Cardiac damage				
NT-pro-BNP (ng/L)	530 (240-1,882)	222 (82-632)	463 (247-1,710)	1,376 (590-3,550)
Increased TnT (≥0.01 ng/mL)	84 (22.4)	13 (14.8)	30 (17.2)	41 (36.3)
Endothelial function				
ADMA (μmol/L)	0.52 (0.45-0.58)	0.49 (0.46-0.57)	0.52 (0.44-0.58)	0.55 (0.47-0.62)
Arginine (mg/dL)	3.1 (2.6-3.5)	3.0 (2.6-3.5)	3.1 (2.7-3.5)	3.1 (2.6-3.5)
vWF (IU/dL)	142 ± 31	134 ± 31	142 ± 32	148 ± 30
Lipids				
Total cholesterol (mg/dL)	217 ± 49	221 ± 42	222 ± 55	207 ± 42
HDL cholesterol (mg/dL)	49 ± 16	49 ± 15	50 ± 16	46 ± 16
Inflammation & nutrition				
C-Reactive protein (mg/L)	4.36 (1.36-9.54)	3.78 (1.06-9.17)	4.93 (1.52-9.74)	4.05 (1.31-9.70)
Albumin (g/dL)	4.2 ± 0.4	4.2 ± 0.4	4.2 ± 0.5	4.1 ± 0.4
Fibrinogen (mg/dL)	348 ± 79	338 ± 77	344 ± 85	363 ± 72
IL-6 (ng/L)	3.9 (2.0-7.9)	3.6 (1.5-7.0)	3.8 (2.2-8.5)	4.4 (2.3-7.1)
TNF-α (ng/L)	17.0 ± 5.4	14.9 ± 4.9	16.7 ± 5.1	19.1 ± 5.4
Calcium and phosphate homeostasis				
Calcium (mg/dL)	9.4 (9.1-9.7)	9.4 (9.2-9.7)	9.3 (9.1-9.7)	9.3 (8.8-9.9)
Phosphorus (mg/dL)	4.2 (3.7-4.9)	3.7 (3.3-4.1)	4.1 (3.6-4.6)	5.3 (4.4-6.3)
25 Hydroxyvitamin D_3_ (ng/mL)	17.8 (11.6-23.3)	20.7 (14.9-27.6)	17.4 (12.1-23.1)	15.4 (10.0-21.4)
1,25 Dihydroxyvitamin D_3_ (pg/mL)	23.9 (16.8-31.6)	30.5 (23.0-38.5)	23.9 (17.0-30.0)	18.7 (13.9-27.4)
Intact PTH (pg/mL)	134 (73-226)	77 (52-135)	144 (89-222)	194 (69-343)
Whole PTH (pg/mL)	74 (42-129)	46 (33-67)	80 (51-121)	102 (38-174)
B Vitamins				
Serum folate (ng/mL)	7.3 (5.2-11.5)	6.6 (4.9-9.2)	7.2 (4.9-11.6)	8.0 (5.6-12.8)
Red cell folate (ng/mL)	214 (165-304)	207 (159-294)	218 (156-293)	223 (175-392)
Vitamin B_12_ (pg/mL)	451 (333-591)	435 (335-587)	451 (334-571)	457 (327-637)
Homocysteine (mg/L)	2.74 (2.14-3.49)	2.28 (1.73-2.80)	2.81 (2.27-3.41)	3.24 (2.45-4.33)
Rheologic markers				
Soluble P selectin (μg/L)	55.0 (45.0-75.0)	53.0 (45.0-70.5)	55.5 (44.5-78.0)	60.0 (48.0-75.0)
Hemoglobin (g/dL)	12.1 ± 2.0	13.6 ± 1.8	12.1 ± 1.8	10.8 ± 1.6

*Note:* Values expressed as mean ± standard deviation, median (25th-75th percentiles), or number (percentage). Missing data: HDL cholesterol (20%), UACR (19%), intact PTH (15%), red cell folate (13%), 1,25 dihydroxyvitamin D_3_ (12%), whole PTH (11%), fibrinogen (11%), and all other markers (≤10% and mostly <5%). Conversion factors for units: creatinine in mg/dL to μmol/L, ×88.4; UACR in mg/g to mg/mmoL, ×0.115; urea in mg/dL to mmol/L, ×0.1667; urate in mg/dL to μmol/L, ×59.48; arginine in mg/dL to μmol/L, ×57.4; cholesterol in mg/dL to mmol/L, ×0.02586; fibrinogen in mg/dL to g/L, ×0.01; calcium in mg/dL to mmol/L, ×0.2495; phosphorus in mg/dL to mmol/L, ×0.3232; 25 hydroxyvitamin D_3_ in ng/mL to nmol/L, ×2.496; 1,25 dihydroxyvitamin D_3_ in pg/mL to pmol/L, ×2.6; PTH in pg/mL to pmol/L, ×0.1073; folate in ng/mL to nmol/L, ×2.266; vitamin B_12_ in pg/mL to pmol/L, ×0.738; and homocysteine in mg/L to μmol/L, ×7.397. No conversion necessary for troponin T in ng/mL and μg/L. Abbreviations: ADMA, asymmetric dimethylarginine; CKD, chronic kidney disease; CRIB, Chronic Renal Impairment in Birmingham; eGFR, estimated glomerular filtration rate; HDL, high-density lipoprotein; K/DOQI, Kidney Disease Outcomes Quality Initiative; IL-6, interleukin 6; MDRD, Modification of Diet in Renal Disease; NT-pro-BNP, N-terminal pro-brain natriuretic peptide; PTH, parathyroid hormone; TNF-α, tumor necrosis factor α; TnT, troponin T; UACR, urinary albumin-creatinine ratio; vWF, von Willebrand factor.

a CKD stage was as defined by the National Kidney Foundation's K/DOQI Work Group in 2002: stage 3, eGFR of 30-60 mL/min/1.73 m^2^; stage 4, eGFR of 15-30 mL/min/1.73 m^2^; and stage 5, eGFR <15 mL/min/1.73 m^2^. eGFR was calculated using the 4-variable MDRD Study equation. Individuals receiving renal replacement therapy at baseline (either dialysis or a kidney transplant) were excluded from the study.

**Table 3:** Incidence of ESRD and Death in the CRIB Cohort

	All	CKD Stage at Baseline^a^		
		III	IV	V
No. of people	382	88	178	116
ESRD				
Total person-years of follow-up	1,571	560	837	173
ESRD	190 (12.1%/y)	9 (1.6%/y)	80 (9.6%/y)	101 (58.2%/y)
Mortality				
Total person-years of follow-up	2,302	590	1,072	640
Vascular	74	10	39	25
Nonvascular	71	11	27	33
Unknown	5	2	2	1
All-cause mortality	150 (6.5%/y)	23 (3.9%/y)	68 (6.3%/y)	59 (9.2%/y)

Abbreviations: CKD, chronic kidney disease; CRIB, Chronic Renal Impairment in Birmingham; eGFR, estimated glomerular filtration rate; ESRD, end-stage renal disease; K/DOQI, Kidney Disease Outcomes Quality Initiative; MDRD, Modification of Diet in Renal Disease.

a CKD stage was as defined by the National Kidney Foundation's K/DOQI Work Group in 2002: stage 3, eGFR of 30-60 mL/min/1.73 m^2^; stage 4, eGFR of 15-30 mL/min/1.73 m^2^; and stage 5, eGFR <15 mL/min/1.73 m^2^. eGFR was calculated using the 4-variable MDRD Study equation. Individuals receiving renal replacement therapy at baseline (either dialysis or a kidney transplant) were excluded from the study.

**Table 4:** Joint Relevance of Baseline Characteristics to ESRD and Death in the CRIB Cohort

Risk Model	*df*	Improvement in Fit Compared With No Markers^a^	C Statistic (95% CI)
**Prediction of ESRD**			
Initial and final selected risk model			
Age, sex and all factors listed in Table S1	22	379.6	0.903 (0.872-0.935)
ln creatinine, ln phosphate, ln UACR, and sex	4	353.6	0.873 (0.836-0.909)
Incremental relevance of factors in final model			
ln creatinine	1	308.5	0.843 (0.804-0.883)
ln creatinine and ln phosphate	2	329.8	0.855 (0.817-0.893)
ln creatinine, ln phosphate, and ln UACR	3	346.1	0.865 (0.828-0.903)
ln creatinine, ln phosphate, ln UACR, and sex	4	353.6	0.873 (0.836-0.909)
**Prediction of All-Cause Mortality**			
Initial and final selected risk model			
Age, sex and all factors listed in Table S2	21	160.8	0.855 (0.813-0.896)
ln NT-pro-BNP, age, current smoking, and increased TnT	4	138.8	0.820 (0.774-0.866)
Incremental relevance of factors in final model			
ln NT-pro-BNP	1	76.1	0.736 (0.684-0.789)
ln NT-pro-BNP and age	2	114.6	0.787 (0.739-0.836)
ln NT-pro-BNP, age, and current smoking	3	129.1	0.811 (0.764-0.857)
ln NT-pro-BNP, age, current smoking, and increased TnT	4	138.8	0.820 (0.774-0.866)

Abbreviations: CI, confidence interval; CRIB, Chronic Renal Impairment in Birmingham; *df*, degrees of freedom (ie, number of terms in model); ESRD, end-stage renal disease; ln, natural logarithm; NT-pro-BNP, N-terminal pro-brain natriuretic peptide; TnT, troponin T; UACR, urinary albumin-creatinine ratio.

a The statistical improvement in model fit between any 2 “nested” models can be evaluated by comparing differences between χ^2^ statistics. For example, for ESRD, the model with all 22 markers is not significantly better at predicting risk than the model containing just log creatinine, log phosphate, log UACR, and sex (because a χ^2^ statistic of 379.6−353.6 = 26 yields *P* = 0.10 when tested against a χ^2^ distribution with 18 *df* [the difference in the number of markers in the 2 nested models]). In comparison, adding log phosphate to a model that contains only log creatinine significantly improves risk prediction for ESRD (because a χ^2^ statistic of 329.8−308.5 = 21.3 yields *P* < 0.001 when tested against a χ^2^ distribution with 1 *df*).

**Table 5:** Adjusted Relative Risks for ESRD and Death in the CRIB Cohort

Model	Baseline Measurement	Comparison	RR (95% CI)
ESRD	Creatinine	Per 50% higher level^a^	3.25 (2.69-3.92)^b^
	Phosphate	Per 30% higher level^a^	1.46 (1.21-1.77)
	ACR	Per 5-fold higher level^a^	1.51 (1.24-1.85)
	Sex	Female vs male	1.54 (1.13-2.09)
Death	Age	Per 15 y older^a^	1.95 (1.54-2.45)
	NT-pro-BNP	Per 5-fold higher level^a^	1.72 (1.41-2.12)
	Cigarette smoking	Current vs not current	2.36 (1.56-3.59)
	TnT	Increased vs not increased^c^	1.83 (1.26-2.66)

Abbreviations: ACR, albumin-creatinine ratio; CI, confidence interval; CRIB, Chronic Renal Impairment in Birmingham; ESRD, end-stage renal disease; NT-pro-BNP, N-terminal pro-brain natriuretic peptide; RR, relative risk; SD, standard deviation; TnT, troponin T.

a Comparisons correspond to about 1-SD differences in age and about 1-SD differences in log creatinine, log phosphate, log ACR, and log NT-pro-BNP (for comparability between characteristics).

b For creatinine, associations with ESRD risk were approximately twice as strong in the earlier years of follow-up than in the later years. Per 50% higher baseline creatinine concentration, the RR of ESRD was 4.71 (95% CI, 3.52-6.30) in the first 2 years of follow-up and 2.43 (95% CI, 1.90-3.10) subsequently. The RR shown therefore is the average RR associated with baseline creatinine level during follow-up.

c Increased defined as ≥0.01 ng/mL.

## Supplementary Materials

Supplementary Table S1 (PDF) Relevance of baseline characteristics to ESRD in the CRIB cohort, given age and sex.

Supplementary Table S2 (PDF) Relevance of baseline characteristics to death in the CRIB cohort, given age and sex.

Supplementary Table S3 (PDF) Baseline characteristics and outcome in the East Kent validation cohort.

Supplementary Table S4 (PDF) Age- and sex- adjusted correlations between those continuous markers found to predict either ESRD or death in the CRIB cohort.
