# Supplementary material for: Prediction of ESRD and Death Among People With CKD: The Chronic Renal Impairment in Birmingham (CRIB) Prospective Cohort Study
Source: Am J Kidney Dis. 2010 Dec;56(6-2):1082–94. doi: 10.1053/j.ajkd.2010.07.016 (PMC2991589; doi:10.1053/j.ajkd.2010.07.016)
Supplement: Supplementary Table S1 (PDF) — Relevance of baseline characteristics to ESRD in the CRIB cohort, given age and sex. [file mmc1.pdf]

Supplementary Table S1; Landray et al, AJKD, “Prediction of ESRD and Death Among People With CKD: The Chronic Renal Impairment in Birmingham (CRIB) Prospective Cohort Study”

Table S1: Relevance of baseline characteristics to ESRD in the CRIB cohort, given age and sex

	Linear model		Quadratic model	
**Baseline characteristic**	**Direction (linear term)**	**Improvement in fit**	**Direction (quadratic term)**	**Improvement in fit**
Ln creatinine	++++	314.5		2.0
Ln cystatin C	++++	201.9		0.6
Urea	++++	195.4		1.6
Ln symmetric dimethylarginine	++++	175.8		2.7
Ln phosphate	++++	139.5	----	28.7
Haemoglobin	----	96.9		1.5
Ln N-terminal pro-B-type natriuretic peptide	++++	86.1		1.7
Ln urinary albumin:creatinine ratio	++++	55.9		3.9
Ln whole parathyroid hormone	++++	37.2		4.7
Ln 1,25 dihydroxy vitamin D3	----	36.8		0.6
Ln intact parathyroid hormone	++++	36.6	++	7.5
Ln homocysteine	++++	23.5		0.0
Ln asymmetric dimethylarginine	++++	23.3		0.2
Elevated troponin T (>=0.01 ug/L)	++++	19.3		.
Tumour necrosis factor-alpha	++++	18.7		5.0
Albumin	----	17.0		1.7
Ln calcium	---	13.8	++++	19.5
Ln 25 hydroxy vitamin D3	---	12.6		0.1
Total cholesterol	--	9.8		0.0
Fibrinogen	++	8.0		0.6

Ln = Natural logarithm. +/- indicates the direction of the association (the number of symbols indicates the extent of statistical significance: two [p<0.01]; three [p<0.001]; four [p<0.0001]).
* Under the null hypothesis of no association, the 'improvement in fit' for each characteristic follows a chi square distribution with 1 degree of freedom. Thus, values >6.6 are significant at the p=0.01 level and values >10.8 are significant at the p=0.001 level. In total, 44 baseline characteristics were assessed for their predictive value in age- and sex-adjusted analyses, of which the above were all significant at the p=0.01 level (either in linear models [tested against one degree of freedom] or in quadratic models [tested against 2 degrees of freedom]).
