# Supplementary material for: Prediction of ESRD and Death Among People With CKD: The Chronic Renal Impairment in Birmingham (CRIB) Prospective Cohort Study
Source: Am J Kidney Dis. 2010 Dec;56(6-2):1082–94. doi: 10.1053/j.ajkd.2010.07.016 (PMC2991589; doi:10.1053/j.ajkd.2010.07.016)
Supplement: Supplementary Table S2 (PDF) — Relevance of baseline characteristics to death in the CRIB cohort, given age and sex. [file mmc2.pdf]

Supplementary Table S2; Landray et al, AJKD, “Prediction of ESRD and Death Among People With CKD: The Chronic Renal Impairment in Birmingham (CRIB) Prospective Cohort Study”

Table S2: Relevance of baseline characteristics to death in the CRIB cohort, given age and sex

	Linear model		Quadratic model	
**Baseline characteristic**	**Direction (linear term)**	**Improvement in fit**	**Direction (quadratic term)**	**Improvement in fit**
Ln N-terminal pro-B-type natriuretic peptide	++++	46.7		0.1
Ln cystatin C	++++	33.5		4.5
Elevated troponin T (>=0.01 ug/L)	++++	29.8		.
Ln symmetric dimethylarginine	++++	25.7		0.3
Ln creatinine	++++	23.0		2.6
Urea	++++	19.1		1.7
Ln 25 hydroxy vitamin D3	----	16.0		0.6
Current cigarette smoker	+++	15.1		.
Ln urinary albumin:creatinine ratio	+++	15.0		2.0
Albumin	---	14.6	----	15.2
Haemoglobin	---	13.4		1.2
Ln 1,25 dihydroxy vitamin D3	---	11.3		2.3
Fibrinogen	++	10.3		0.0
Ln asymmetric dimethylarginine	++	10.2		0.1
Ln C-reactive protein	++	9.7		0.0
Tumour necrosis factor-alpha	++	9.1		1.7
Ln phosphate	++	8.7		4.7
Ln calcium	--	8.3		3.6
Ln intact parathyroid hormone		5.0	++	7.3

Ln = Natural logarithm. +/- indicates the direction of the association (the number of symbols indicates the extent of statistical significance: two [p<0.01]; three [p<0.001]; four [p<0.0001]).
* Under the null hypothesis of no association, the 'improvement in fit' for each characteristic follows a chi square distribution with 1 degree of freedom. Thus, values >6.6 are significant at the p=0.01 level and values >10.8 are significant at the p=0.001 level. In total, 44 baseline characteristics were assessed for their predictive value in age- and sex-adjusted analyses, of which the above were all significant at the p=0.01 level (either in linear models [tested against one degree of freedom] or in quadratic models [tested against 2 degrees of freedom]).
