# Supplementary material for: Prediction of ESRD and Death Among People With CKD: The Chronic Renal Impairment in Birmingham (CRIB) Prospective Cohort Study
Source: Am J Kidney Dis. 2010 Dec;56(6-2):1082–94. doi: 10.1053/j.ajkd.2010.07.016 (PMC2991589; doi:10.1053/j.ajkd.2010.07.016)
Supplement: Supplementary Table S3 (PDF) — Baseline characteristics and outcome in the East Kent validation cohort. [file mmc3.pdf]

Table S3: Baseline characteristics and outcome in the East Kent validation cohort

		**CKD stage at baseline**[a]		
	**All**	**III**	**IV**	**V**
Number of people	213	55	66	92
Age, years	65.1 (13.5)	65.7 (14.0)	68.8 (11.0)	62.1 (14.4)
Estimated GFR[b], ml/min/1.73m^2	21.6 (13.6)	40.6 (7.7)	22.3 (5.6)	9.6 (2.6)
Number (%) of men	137 (64.3%)	42 (76.4%)	45 (68.2%)	50 (54.3%)
Disease history				
Vascular disease	92 (43.2%)	30 (54.5%)	35 (53.0%)	27 (29.3%)
Diabetes Mellitus	57 (26.9%)	11 (20.4%)	24 (36.4%)	22 (23.9%)
Left Ventricular Hypertrophy[c]	123 (64.1%)	28 (51.9%)	38 (64.4%)	57 (72.2%)
Current cigarette smoker	20 (9.4%)	4 (7.3%)	6 (9.1%)	10 (10.9%)
Physical measurements				
Body mass index, kg/m^2	28.6 (6.2)	29.5 (5.6)	28.8 (6.3)	27.8 (6.5)
SBP, mmHg	139.6 (16.9)	135.2 (16.4)	139.2 (19.0)	142.6 (15.0)
DBP, mmHg	77.4 (9.0)	76.4 (7.9)	77.7 (10.1)	77.7 (9.0)
Laboratory measures				
Creatinine, mg/dL	3.3 (2.1 - 5.2)	1.7 (1.6 - 1.9)	2.9 (2.5 - 3.3)	5.7 (4.9 - 6.9)
Phosphorus, mg/dL	4.0 (3.6 - 4.7)	3.6 (3.2 - 3.9)	3.7 (3.4 - 4.1)	4.8 (4.1 - 5.6)
NT-pro brain natriuretic peptide, pg/mL	757 (264 - 2058)	281 (111 - 740)	578 (264 - 1139)	1637 (604 - 3422)
Number with elevated troponin T (>=0.01 ng/mL)	91 (42.7%)	11 (20.0%)	25 (37.9%)	55 (59.8%)
Incidence of ESRD and all-cause mortality				
ESRD	66 (12.1% p.a.)	0 (0.0% p.a.)	6 (3.1% p.a.)	60 (37.2% p.a.)
All-cause mortality	65 (9.2% p.a.)	12 (6.4% p.a.)	21 (9.9% p.a.)	32 (10.4% p.a.)

Mean (SD), median (IQR) or n (%) shown.
SBP=Systolic blood pressure; DBP=Diastolic blood pressure; ESRD=End-Stage Renal Disease
[a] CKD stage as defined by the National Kidney Foundation K/DOQI Work Group (2002)
[b] Estimated using the simplified Modification of Diet in Renal Disease (MDRD) equation
[c] Based on echocardiography
