# Supplementary material for: Prediction of ESRD and Death Among People With CKD: The Chronic Renal Impairment in Birmingham (CRIB) Prospective Cohort Study
Source: Am J Kidney Dis. 2010 Dec;56(6-2):1082–94. doi: 10.1053/j.ajkd.2010.07.016 (PMC2991589; doi:10.1053/j.ajkd.2010.07.016)
Supplement: Supplementary Table S4 (PDF) — Age- and sex- adjusted correlations between those continuous markers found to predict either ESRD or death in the CRIB cohort. [file mmc4.pdf]

Supplementary Table S4; Landray et al, AJKD, “Prediction of ESRD and Death Among People With CKD: The Chronic Renal Impairment in Birmingham (CRIB) Prospective Cohort Study”

Table S4: Age- and sex- adjusted correlations between those continuous markers found to predict either ESRD or death in the CRIB cohort

**Biomarker**	**Creatinine**	**Cystatin C**	**Urea**	**ADMA**	**SDMA**	**ACR**	**Calcium**	**Phosphate**	**Haemoglobin**	**NT-proBNP**	**Albumin**	**HCY**	**TC**	**25-D3**	**1,25-D3**	**Fibrinogen**	**TNFA**	**IPTH**	**WPTH**
Cystatin C	**0.85**	1																	
Urea	**0.82**	**0.77**	1																
ADMA	0.21	0.32	0.20	1															
SDMA	**0.82**	**0.82**	**0.70**	0.33	1														
ACR	0.39	0.40	0.33	-	0.34	1													
Calcium	-	-	-	-	-	-	1												
Phosphate	**0.68**	**0.65**	**0.69**	-	**0.57**	0.37	-	1											
Haemoglobin	-	-	-	-	-	-	-	-	1										
NT-proBNP	0.48	**0.53**	0.44	0.23	**0.53**	0.37	-	0.38	-	1									
Albumin	-	-	-	-	-	-	0.39	-	0.24	-	1								
Homocysteine	0.36	0.42	0.34	-	0.43	-	-	-	-	-	0.22	1							
Total cholesterol	-	-	-	-	-	-	-	-	-	-	-	-	1						
25 hydroxy vitamin D3	-	-	-	-	-	-	-	-	0.23	-	0.26	-	-	1					
1,25 dihydroxy vitamin D3	-	-	-	-	-	-	0.20	-	0.34	-	0.23	-	-	0.23	1				
Fibrinogen	-	0.20	-	-	-	-	-	-	-	-	-	-	-	-	-	1			
TNFA	0.29	0.41	0.20	0.20	0.30	-	-	-	-	-	-	-	-	-	-	-	1		
IPTH	0.26	0.32	0.20	-	0.31	-	-	-	-	0.29	-	-	-	-	-	-	-	1	
WPTH	0.22	0.26	-	-	0.28	-	-	-	-	0.24	-	-	-	-	-	-	-	**0.98**	1
CRP	-	-	-	-	-	-	-	-	-	-	-	-	-	-	-	0.30	0.26	-	-

ADMA=Asymmetric dimethylarginine; SDMA=Symmetric dimethylarginine; ACR=Urinary albumin:creatinine ratio; HCY=Homocysteine; TC=Total cholesterol; 25-D3=25 hydroxy vitamin D3; 1,25-D3=1,25 dihydroxy vitamin D3; TNFA=Tumor necrosis factor alpha; IPTH=Intact parathyroid hormore; WPTH=Whole parathyroid hormone; CRP=C-reactive protein.
'-' = The magnitude of the correlation is <0.2.
